# A systematic scoping review of the molecular pathogenesis of digestive system cancers (2013–2025)

**DOI:** 10.1007/s12672-026-05374-6

**Published:** 2026-06-19

**Authors:** Shirin Hui Tan, Sivasangari Subramaniam, Wei Hong Lai, Farah Razali, Lee Len Tiong, Pei Jye Voon, Edmund Ui-Hang Sim

**Affiliations:** 1 Clinical Research Centre, Sarawak General Hospital, Ministry of Health Malaysia, Jalan Hospital, 93586 Kuching, Sarawak, Malaysia; 2 Clinical Research Centre, Hospital Pulau Pinang, Ministry of Health Malaysia, Jalan Residensi, 10990 Georgetown, Pulau Pinang, Malaysia; 3 Department of Radiotherapy, Oncology and Palliative Care, Sarawak General Hospital, Jalan Hospital, 93586 Kuching, Sarawak, Malaysia; 4 Institute for Clinical Research, National Institutes of Health, Ministry of Health Malaysia, Persiaran Setia Murni, Setia Alam, 40170 Shah Alam, Selangor Malaysia; 5 Faculty of Resource Science and Technology, Universiti Malaysia Sarawak, 94300 Kota Samarahan, Sarawak, Malaysia

**Keywords:** Digestive system neoplasm, Molecular pathogenesis, Next-generation sequencing, Precision medicine, Mutation

## Abstract

Digestive system cancers (DSCs) remain among the most prevalent and lethal malignancies worldwide, accounting for more than a quarter of cancer diagnoses and a third of cancer deaths. Advances in next-generation sequencing (NGS) have accelerated discovery of genetic, epigenetic, and transcriptomic alterations, yet an integrated overview across DSCs is lacking. This scoping review synthesized 57 studies published between 2013 and 2025 from PubMed, Scopus, and Web of Science. Colorectal and gastric cancers were most frequently represented, while oesophageal, pancreatic, and rare subtypes such as gallbladder and ampullary cancers were under-studied. Recurrent alterations in *TP53*, *KRAS*, *PIK3CA*, and *CDKN2A* were identified, alongside mismatch repair deficiency and microsatellite instability in colorectal and gastric cancers. Emerging evidence from recently published studies further highlights the contribution of germline alterations and increased molecular heterogeneity in selected DSC subtypes. Epigenetic dysregulation and recent applications of single-cell and spatial transcriptomics highlighted tumour heterogeneity. Despite progress, evidence remains uneven across cancer types, with limited exploration of germline variants beyond colorectal, pancreatic, and select liver cancers.

**Supplementary Information:**

The online version contains supplementary material available at https://doi.org/10.1007/s12672-026-05374-6.

## Introduction

Digestive system cancers (DSCs) represent a biologically and clinically diverse group of malignancies originating from the gastrointestinal tract and associated organs, including the oesophagus, stomach, colorectal region, liver, and pancreas [1]. Together, these cancers account for more than 25% of all cancer diagnoses and contribute to approximately 35% of global cancer-related deaths [2]. Among them, liver, colorectal, and gastric cancers rank within the top ten most commonly diagnosed cancers, highlighting the substantial global health burden posed by DSCs. Despite notable advancements in therapeutic strategies, overall five-year survival rates remain below 20%, largely due to late-stage diagnoses and the intrinsically aggressive nature of these malignancies [2–4].

A key contributor to this poor prognosis is the frequently asymptomatic or non-specific clinical presentation of DSCs during their early stages, which hampers timely detection and intervention [4, 5]. While well-established risk factors such as diet, lifestyle, chronic infections, and environmental exposures have long been recognised, emerging evidence has underscored the pivotal role of genetic and molecular alterations in driving carcinogenesis, disease progression, and treatment resistance across DSC subtypes [5, 6]. Although progress in imaging technologies and endoscopic screening has improved early detection in certain populations, molecular biomarkers with diagnostic, prognostic, or therapeutic utility have yet to be fully integrated into clinical workflows. The translational gap between molecular discoveries and their routine application remains particularly pronounced in rare DSC subtypes and late-stage disease settings [2, 5, 6].

Understanding the molecular pathogenesis of DSCs namely, the biological mechanisms underlying tumour initiation and progression is increasingly recognised as critical to informing precision oncology efforts. The development of these malignancies results from a complex interplay of somatic mutations, germline predisposition, and environmental factors. While hereditary cancer syndromes such as Lynch syndrome and familial adenomatous polyposis explain approximately 5% of DSCs, a broader hereditary contribution is suspected in up to 20–25% of cases [7, 8]. Advances in genome-wide association studies (GWAS) and biobank-driven analyses have revealed multiple susceptibility loci and regulatory variants linked to gastrointestinal cancers, particularly colorectal cancer [9, 10]. These insights have begun to delineate both overlapping and distinct genetic architectures across DSC subtypes. However, despite significant progress in identifying genetic risk factors and their associations with treatment outcomes, comparatively few studies have examined the underlying molecular mechanisms that drive disease development and therapeutic resistance.

This persistent gap in understanding calls for a comprehensive synthesis of the molecular landscape of DSCs. Consolidating current evidence is essential to identify commonly altered molecular pathways, highlight underrepresented cancer types, and pinpoint critical areas for future research. Bridging this knowledge will not only enhance biological insight but also accelerate the translation of molecular findings into more precise and effective diagnostic and therapeutic interventions.

To address this need, the present scoping review was conducted using the Arksey and O’Malley framework [11], in accordance with the PRISMA-ScR (Preferred Reporting Items for Systematic Reviews and Meta-Analyses extension for Scoping Reviews) guidelines [12]. This review systematically examines literature published between 2013 and 2025, mapping the molecular basis of DSCs with a focus on key genetic mutations, dysregulated signalling pathways, and molecular alterations across cancer subtypes. By synthesising the existing body of evidence, we aim to identify significant research gaps, underexplored tumour entities, and potential molecular targets. This work ultimately seeks to inform research priorities and support the integration of molecular evidence into clinical practice, thereby advancing the goals of precision oncology in the context of DSCs.

## Methods

This scoping review was conducted in accordance with the methodological framework proposed by Arksey and O’Malley [11] with enhancements guided by the Joanna Briggs Institute (JBI) Manual for Evidence Synthesis [12]. Reporting conforms to the PRISMA-ScR (Preferred Reporting Items for Systematic Reviews and Meta-Analyses Extension for Scoping Reviews) checklist to ensure transparency and reproducibility [13]. The review process was structured across five key stages: 1. identifying the research question, 2. identifying relevant studies, 3. study selection, 4. charting the data, and 5. collating, summarising, and reporting the results. Although some frameworks recommend a consultation exercise to enhance interpretation and identify additional sources, this step was not undertaken due to time constraints [14]. Clinical trial number: not applicable.

### Identifying the research question

The review aimed to synthesise recent literature on the molecular pathogenesis of DSCs, with an emphasis on the use of next-generation sequencing (NGS) technologies. The temporal scope was limited to publications from 2013 to 2025 to capture developments in high-throughput sequencing methodologies and the most recent advances in molecular characterisation. To maintain breadth while allowing focused analysis, the following research questions were developed:

- Q1: How many studies employing NGS for DSCs were published between 2013 and 2025?
- Q2: What are the most frequently studied genetic mutations identified through NGS in DSCs during this period, and how have research foci on these mutations evolved over time?

### Identifying relevant studies

A comprehensive literature search was conducted on January 27, 2025, across three electronic databases: PubMed/MEDLINE, Scopus, and Web of Science. The search strategy was subsequently updated on April 13, 2026 to include studies published up to 2025 prior to final data synthesis. The search strategy employed both controlled vocabulary (e.g., MeSH terms) and free-text keywords, including but not limited to: “gastrointestinal cancer,” “digestive system neoplasm,” “colorectal cancer,” “gastric cancer,” “oesophageal cancer,” “liver cancer,” “pancreatic cancer,” “next-generation sequencing,” “molecular pathology,” and “genetic changes” [11, 13]. The complete search strategy is detailed in Supplementary Methods. Filters were applied to limit studies to those published in English and with full-text availability.

Eligible study designs primarily included original research articles such as cross-sectional, cohort, and case–control studies that investigated molecular mechanisms in digestive system cancers using next-generation sequencing (NGS). Review articles were also included where relevant to provide contextual understanding and to summarise broader evidence. Following an updated literature search to include studies published up to 2025, additional eligible studies were incorporated to ensure that the review reflects the most current evidence on molecular alterations in digestive system cancers. While formal risk-of-bias assessment was not undertaken due to the scoping nature of this review, systematic reviews were retained for their value in summarising high-level evidence and identifying research gaps.

### Study selection

Search results were imported into Microsoft Excel 2016, where duplicate records were removed. Title and abstract screening were performed independently by two reviewers (SHT, SS) using pre-specified inclusion and exclusion criteria. Discrepancies were resolved by a third reviewer (WHL). Articles that met criteria for full-text review were assessed in duplicate by the same reviewers, with any disagreements again adjudicated by consensus or third-party consultation [11, 14].

Key variables extracted included: publication year, study design, cancer subtype, NGS methodology, identified molecular markers, data sources (e.g., genomic repositories), study outcomes, and recommendations for future research. The final set of included studies was reviewed and approved by all co-authors. Where possible, included studies were reviewed to minimise overlap in study populations. No significant duplication of cohorts was identified based on available study descriptions. In addition, this scoping review was designed to include studies across a broad spectrum of digestive system cancers to capture both shared and tumour-specific molecular alterations, while interpretation was performed within both cross-cancer and tumour-specific contexts to preserve cancer-type-specific insights.

### Charting the data

Data extraction was guided by a structured framework comprising predefined variables, developed based on prior scoping review methodologies and domain specific literature. The framework was pilot-tested by two reviewers on a subset of studies to ensure consistency and relevance [11, 14]. Discrepancies in interpretation were resolved through discussion or consultation with a third reviewer, and modifications to the framework were made accordingly [11, 14]. Studies were categorised by DSCs subtype, including colorectal cancer (CRC), gastric cancer, liver cancer, pancreatic cancer, oesophageal cancer, and other DSCs. Extracted data were compiled into a Microsoft Excel 2016 spreadsheet for thematic coding and descriptive statistical analysis. A summary of extracted study characteristics is presented in Table 2, while the synthesis of molecular pathways and genetic alterations is summarised in Table 3.

### Collating, summarising, and reporting the results

The final phase involved synthesising the extracted data to address the research questions and identify patterns across studies. Narrative and descriptive summaries were developed to highlight the most frequently studied mutations, dominant molecular pathways, and temporal trends in NGS applications to DSCs. Thematic analysis was used to categorise findings according to cancer subtype and molecular target. In addition to tabulation, a qualitative visual synthesis of recurrently reported genes and pathways across cancer types (Fig. 2) was developed to facilitate cross-cancer comparison while preserving tumour-specific context.

This scoping review is registered with the National Medical Research Register (NMRR ID-23–02053-JLS) and received an exemption from ethics review by the Medical Research & Ethics Committee (MREC), as it did not involve human participants.

## Results

### Study selection

**Fig. 1:**
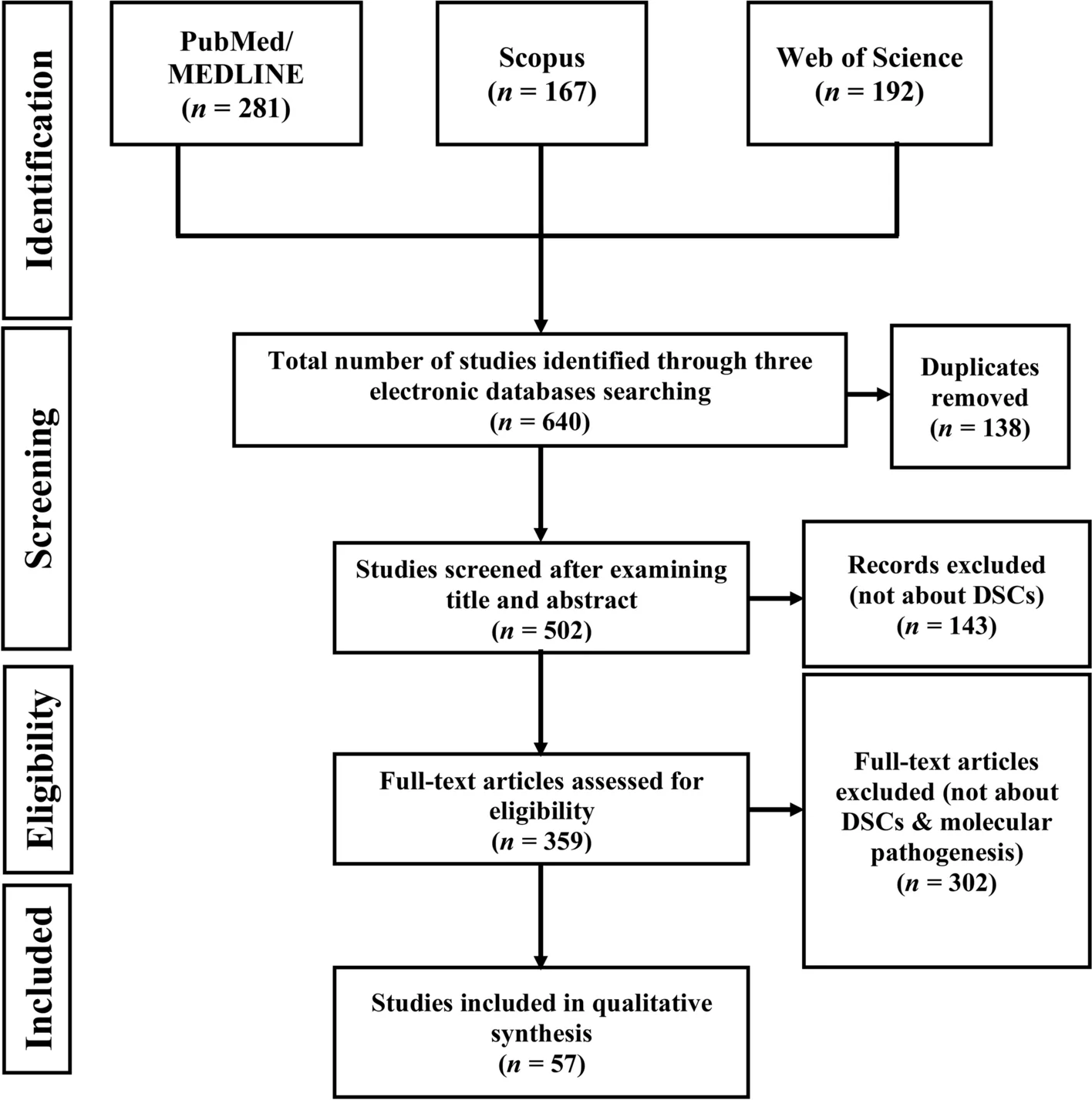
Preferred reporting items for systematic review and meta-analyses (PRISMA) flow diagram for scoping review on the landscape of molecular pathogenesis in DSC

A total of 640 records were identified through database searches. After removing 138 duplicates, 502 unique records were screened by title and abstract. Of these, 143 articles were excluded for being unrelated to DSCs. Full-text assessments were conducted on the remaining articles, and additional exclusions were made based on eligibility criteria. An overview of the publication years, article types, and cancer subtypes covered by the included studies is presented in Table 1. Ultimately, 57 studies that investigated the molecular pathogenesis of DSCs were included in this review, comprising of 46 original research articles and 11 review articles. The study selection process is illustrated in the PRISMA flow diagram (Fig. 1).

**Table 1:** Overview of the published journals analysed in this scoping review

Items	Number of articles, N (%)
Year published	
2013–2017	19
2018–2025	38
Types of articles	
Original research	46
Systematic review	11
Others (e.g. opinion, short-comm, etc.)	0
Types of DSC	
Oesophagus	2
Stomach/gastric	13
Liver	9
Pancreas	9
Colon	19
Rectum	3
Others	2

### Study characteristics

The selected studies spanned multiple geographic regions, with the largest contributions from China (n = 15) and the United States (n = 9). Other studies originated from Korea (n = 4), Germany, Italy, and Japan (n = 3 each), the Netherlands, Poland, India, and Slovenia (n = 2 each), and one study each from Switzerland, the United Kingdom, Spain, Russia, Saudi Arabia, Iran, France, Denmark, and Australia. Two studies were multinational in nature, while two did not specify a location.

In terms of study design, the majority of included studies (n = 46) were original research articles employing cross-sectional, retrospective, or cohort-based methodologies, while a smaller proportion comprised systematic or narrative reviews (n = 11). Most studies utilized next-generation sequencing (NGS)-based approaches, including targeted sequencing panels, whole-exome sequencing, and whole-genome sequencing, to investigate molecular alterations in digestive system cancers (Table 2).

**Table 2:** Details of published articles analysed in this scoping review

#	Authors	Title	Cancer	Gene-function(S)	Summary/recommendation/conclusion
1.	Ahadova, A. et al. 2018	Three molecular pathways model colorectal carcinogenesis in Lynch syndrome	Colorectal Cancer	MMR deficiency reported in 76.7% of the adenoma cases from Lynch Syndrome (LS) mutation carriers. Histological and molecular evidence showed *KRAS* and *APC* mutations occur after MMR deficiency onset	This study reconstructed colorectal carcinogenesis in Lynch Syndrome. Collection of molecular and histology evidence from LS adenomas to show that LS-associated colorectal cancers follow three pathways characterized by the type and timing of fundamental mutation events. The study suggested further research on the other pathways
2.	Alatise, O. et al. 2021	Molecular and phenotypic profiling of colorectal cancer patients in West Africa reveals biological insights	Colorectal Cancer	Tumours from Nigerian CRC cases showed lower *APC* mutation frequency but higher WNT pathway alterations compared to reference cohorts. Mutations identified include *TP53* (71.7% in Nigeria vs. 77.5% MSKCC), *KRAS* (56.5% in Nigeria vs. 44.1% in MSKCC), *APC* alteration among Nigeria and MSKCC cohorts (36.9% and 76.0% respectively). The two groups had similar *CTNNB1* gene alterations, but their P13K pathway alterations *PIK3R1* and *PIK3CB* differed; the Nigeria cohort was more likely than the MSKCC cohort to have higher alterations *PIK3R1* (8.7% vs. 2.2%) and *PIK3CB* (4.3% vs. 0.5%)	The study compared the molecular and phenotypic profile of CRC to determine microsatellite stability (MSI) and pathway alterations to determine regional unique CRC biology
3.	Andersen, J. et al. 2015	Molecular pathogenesis of intrahepatic cholangiocarcinoma	Liver cancer	Key signalling pathways in ICC pathogenesis include the presence of epidermal growth receptor *ERBB2*, *EGFR*, and *RAS*, increased *P13K/AKT/mTOR*, hepatocyte growth factor JAK/STAT, and VEGF (vascular endothelial growth factor) pathways	The study discussed ICC pathogenesis and somatic mutations found in the disease
4.	Astaras, C. et al. 2023	The first comprehensive genomic characterization of rectal squamous cell carcinoma	Colorectal Cancer (Rectal)	In-depth genomic analysis has revealed *PIK3CA*, *PTEN*, *TP53*, *ATM*, *BCL6*, *SOX2* mutation. *PI3K/Akt/mTOR* is the most affected pathway in rectal squamous cell carcinoma (rSCC)	The genomic analysis on rSCC samples revealed mutational and copy number variation profiles in rSCC. This analysis shows rSCC molecular profile is similar to aSCC (anal squamous cell carcinoma)
5.	Caretheres, J. et al., 2015	Genetics and genetic biomarkers in sporadic colorectal cancer	Colorectal Cancer (sporadic)	MSI and *POLE* mutations are reported in 15% of sporadic CRC cases and various copy number alterations in 85% of CRC cases that contain *PIK3CA* and *KRAS* oncogenic activation. There is also a loss of heterozygosity of *APC* and *TP53* tumour suppressor genes	Evaluation of genetic biomarkers in sporadic CRC categorized driver gene alterations in the disease and classified them into a hypermutated group of defective DNA mismatch repair, non-hypermutated group, CpG Island Methylator Phenotype, and raised microsatellite alterations. The study suggested further investigation of the role of the microbiome in sporadic CRC
6	Chen, H. et al., 2018	Identification of the potential molecular mechanism and driving mutations in the pathogenesis of familial intestinal gastric cancer by whole exome sequencing	Gastric Cancer (Familial intestinal gastric cancer)	*ESR1* homozygous mutations in exon 1 and exon 10 were reported in most familial intestinal gastric cancer (FIGC) cases. Additionally, ESR1 heterozygous deletion of 68-69GT nucleotides was confirmed through exome sequencing	A pathway, function, and network analysis of overlapped genes bearing insertions-deletions (INDELs) and single-nucleotide variants (SNVs) revealed direct and indirect interactions of hub proteins in the protein–protein interactions
7.	Crumley, S. et al., 2016	Next-generation sequencing of matched primary and metastatic rectal adenocarcinomas demonstrates minimal mutation gain and concordance to colonic adenocarcinomas	Colorectal Cancer (rectal adenocarcinoma	Identical mutations in primary and metastasis tumours were found in most rectal adenocarcinoma cases. These mutations include *SMAD4, PIK3CA, FGFR3, BRAF, GNAS, NRAS, APC, TP53, FBXW7, and KRAS*	The study evaluated the somatic mutations in rectal adenocarcinoma and determined that rectal adenocarcinoma and colonic adenocarcinoma have similar molecular pathogenesis
8.	Din, S. et al., 2018	The mutational analysis identifies therapeutic biomarkers in inflammatory bowel disease–associated colorectal cancers and predictive biomarkers in IBD-associated colorectal cancers	Colorectal Cancer	Hypermutation was observed in 27% of the cases, and TP53, *APC, PIK3CA,* and *KRAS identified* as driver genes in inflammatory bowel disease-associated colorectal cancers (IBD-CRCs). Six signatures identified in the samples include *POLE* mutations, deamination of 5-methylcytosine, AID/APOBEC activation, dMMR (deficient mismatch repair), and MSI	This study analysed therapeutic biomarkers in IBD-CRCs to identify mutational signatures, somatic point mutation, somatic copy number alterations, and small insertions and deletions of genetic variants and suggested further investigation is the germline variations that indicate CRC susceptibility in IBD cases
9.	El Dika, I. et al., 2020	Molecular profiling and analysis of genetic aberrations aimed at identifying potential therapeutic targets in fibrolamellar carcinoma of the liver	Liver Cancer (Fibrolamellar carcinoma)	*DNAJB1-PRKACA* fusion transcript was detected in all the tumour samples. TERT promoter mutation was the second most common genetic aberration. There were mutations in *SF3B1, CTNNB1*and *ZFHX3* genes. Germline mutation also observed in the analysis is *MUTYH*	The study analysed *DNAJB1-PRKACA* fusion and germline sequencing, identified *DNAJB1-PRKACA* as a fusion detectable through DNA and RNS sequencing, and suggested a further study on FLC tumorigenesis
10.	El-Deiry, W. et al., 2015	Molecular profiling of 6,892 colorectal cancer samples suggests different possible treatment options specific to metastatic sites	Colorectal Cancer	*BRAF* mutations were associated with altered protein expression patterns, suggesting molecular differences between metastatic colorectal cancer sites. *KRAS* mutation in metastatic CRC had higher c-MET protein expression than *KRAS* mutation in CRC	Molecular profiling of CRCs cases showed the difference between *BRAF/KRAS* mutations in the primary and metastatic sites, and the difference in the mutations and protein expression requires further research
11.	Fleitas-Kanonnikoff, T.et al. 2019	Molecular profile in Paraguayan colorectal cancer patients, towards a precision medicine strategy	Colorectal Cancer	Gene mutations identified in the analysis include *FGFR, KRAS, BRAF, NRAS, and P13KCA* in 55.6% of the samples, while 14% presented with MSI	The study analysed the molecular profile and microsatellite states of CRC samples and suggested further investigations on MMR genetic analysis of germline mutation of individuals below 50 years with a family history of CRC
12.	Ghorbani, M. et al. 2021	Aberrantly methylated-differentially genes and pathways among Iranian patients with colorectal cancer	Colorectal Cancer	Pathway enrichment analysis identified aberrant methylation in *WNT*, *BMP2*, and *SFRP2* pathways, suggesting epigenetic dysregulation in colorectal carcinogenesis. Additionally, *WNT2, BMP2, SFRP2*, and *ZNF726* node proteins showed close interconnection with other node proteins	The study evaluated aberrantly differentially methylated genes (DMGs) and pathways and determined that hub genes *WNT2, BMP2, SFRP2,* and *ZNF726* are possible CRC diagnostic and therapeutic targets
13.	Hirano, D. et al. 2019	Early-stage serrated adenocarcinomas are divided into several molecularly distinct subtypes	Colorectal Cancer -Serrated adenocarcinoma	The reported findings of colon cancer subtyping (CCS) show a higher frequency of invasive cancer in all cases of colon cancer subtype 3 involving *CDX2*, *FRMD6* + , *HTR2B*, MSI-L/MSS + , and MSI-L/MSS than in CCS1 involving *FRMD6-, HTR2B-, CDX2* + *, ZEB1-* and MSI-L/MSS. Epithelial serration and Ki67 were also high in CCS3 than in CC1. *KRAS* and *BRAF* mutations were also higher in *CC3* than in *CCS1*	The study classified genetic changes and clinicopathological features, SAC epithelial serration, and Ki 67 labelling index (LI) of different types of SAC and proposed a multicentre study with a large number of cases
14.	Hissong, E. et al. 2018	Gastric carcinomas with lymphoid stroma	Gastric Cancer	All tumours showed EBV/MMR classified tumours had PD-L1 expression, multiple tumour infiltrating lymphocytes, and sporadic nuclear ϐ catenin accumulation. Furthermore, they lacked membranous *HER2* amplification and *HRE2* staining. EBV^−^/MMR-deficient tumours had higher mutation burden and *KRAS* alterations than EBV-/MMR-P tumours. Alterations occurred in *KRAS, PIK3CA, ARID1A,* and *TP53* genes	The study classified gastric carcinoma tumours into EBV/MMR-proficient, EBV/MMR deficient, and EBV/MMR-P categories
15.	Ho, D. et al., 2016	Molecular pathogenesis of hepatocellular carcinoma	Liver Cancer	Sequencing data from HCC samples reveals chromosome gains and losses Chromosome gains reported in HCC sample analysis are 1q, 5p, 6p, 8q,17q, 20q, and Xq. Chromosome losses occur at 1p, 4p-q, 6q, 8p, 13p-q, 16p-q, 17p, 21p-q and 22q. Additionally, there was a detection of oncogenesis focal amplifications on *TERT*, *MET, CCND1, MYC*, and *FGF19* gene and tumour suppressor deletions at *TP53*, *CDKN2B*, *PTEN*, and *CDKN2A* Copy number alterations (CNAs)	This study explored molecular alterations, including somatic mutations, copy number alterations, and gene expression, to determine common mutations in *CTNNB1* and *TP53* and identify the high frequency of recurrent mutations in *ARID2*, *CDKN2A*, *MLL*, and *ARID1*. The study suggested further investigation of novel therapies against HCC
16.	Huang, F et al., 2019	Targeted genomic profiling identifies frequent deleterious mutations in *FAT4* and *TP53* genes in HBV-associated hepatocellular carcinoma	Liver Cancer	Sequencing showed 13 non-synonymous mutations: 31% were found in the *TP53* gene, and 69% were in *FAT4*. There were no non-synonymous mutations in *ARID1A*, *PIK3CA*, *HNF4α* & *IRF2*	The study carried out a targeted sequencing of cancer-related genes, including *TP53, PIK3CA, IRF2, ARID1A, FAT4,* and *HNF4α*, and determined the significance of *TP53* and *FAT4* genes in HCC pathogenesis
17.	Huang, J. et al. 2021	*IDH1* and *IDH2* mutations in colorectal cancers	Colorectal Cancer	*IDH1*/*2* was detected in 0.9% of CRC cases and in 3% of the *BRAF*-mutated CRC samples. The study associated three of the *IDH1*/*2* mutations with inflammatory bowel disease	The study explored *ADH1* and *ADH2* mutations in CRCs and suggested further investigation of the clinicopathologic features of *IDH1*/*2-*mutated CRCs
18.	Je, E. et al., 2013	Frameshift mutations of axon guidance genes *ROBO1* and *ROBO2* in gastric and colorectal cancers with microsatellite instability	Gastric & Colorectal Cancers	The coding exons in ROB1 and ROB2 genes had mononucleotide repeats (five frameshift mutations in each gene), which indicates that these repeats are possible targets of MSI cancers. Additionally, the repeats were present in 14.2% of the cases that had high MSI (MSI-H) but not in cases with MSI-L (low MSI)	This study explored somatic mutations and alterations in *ROBO 1* and *ROBO2* genes to determine the possibility of both genes playing a role in CRC and GC pathogenesis
19.	Jesinghaus, M. et al. 2016	Genotyping of colorectal cancer for cancer precision medicine: Results from the IPH Center for Molecular Pathology	Colorectal Cancer	Thirty percent of cases had *BRAF/NRAS* mutations, while *PIK3CA* mutation was in 21% of the cases	The study conducted deep targeted genotyping and analysed MS status, and determined that *BRAF*, *NRAS*, and *PIK3CA* mutations are commonly present in CRC
20.	Kim M. & Seo, A. 2022	Molecular pathology of gastric cancer	Gastric Cancer	EBV-positive GC was reported in 8.8% of GC cases., revealing *MLH1* deficiency and hypermethylation of kinase inhibitor 2A (*CDKN2A*). MSI occurred in 21.7% of the cases, and showed *MLH1* epigenetic silencing and CIMP methylation. Genomically stable (GS) reported in 19.7% of the cases showed *ARID1A*, *RHOA*, and *CDH1* mutations. CIN in GC was reported in 49.8% of the cases and characterized by receptor tyrosine kinase (RTKs) amplifications and *KRAS* mutation	This review examined key genetic alterations and methylation in EBV-associated, MSI, GS, and CIN gastric cancers and determined that the available GC morphological classifications are inadequate to guide precise therapy
21.	Kim, M. et al., 2013	Mutational and expressional analyses of *SPOP*, a candidate tumour suppressor gene, in prostate, gastric, and colorectal cancers	Gastric & Colorectal Cancers	Two *SPOP* protein somatic missense mutations were detected in two of the analysed CRC cases. These mutations were present in the p.Ser14Leu, p.Phe133Leu, and p.Tyr87Cys coding sequences	This study analysed *SPOP* protein expression in GC and mutations in CRC and established that *SPOP* expression is common in CRC and GC, but the not somatic mutation is rare
22.	Kit, O. et al., 2020	Identification of new candidate genes and signalling pathways associated with the development of neuroendocrine pancreatic tumours based on next-generation sequencing data	Pancreatic Cancer (Pancreatic neuroendocrine tumours)	Parallel sequencing revealed 67% missense mutations and 18% activating mutations. Further, 35% of the variants resulted in encoded protein function loss. In 38% of the cases, genetic changes in the SMAD2/3 signalling pathway regulation	This study analysed candidate genes *PRKAR1A, CREB1, BUB1B, BCL11A,* and *TCF12*, and molecular changes in SMAD2/3 signalling pathway and identified genetic features that drive Pancreatic neuroendocrine tumours (PanNETs)
23.	Konukiewitz, B. et al. 2018	Pancreatic neuroendocrine carcinomas reveal a closer relationship to ductal adenocarcinomas than to neuroendocrine tumours G3	Pancreatic Cancer (Pancreatic neuroendocrine tumours)	Most cases showed *TP53* mutations (eight out of the twelve cases), and five cases out of the analysed sample showed *KRAS* mutation. RB1 expression loss was present in four of the cases. Most of the *KRAS*-positive showed ductal differentiation expressed through carcinoembryonic antigen and *MUC1*	This study explored *TP53* and *RB1* alterations to reveal *APC*, *KRAS*, and *BRAF* mutations in most neuroendocrine carcinomas
24.	Lee, S. et al., 2016	Frequent somatic *TERT* promoter mutations and *CTNNB1* mutations in hepatocellular carcinoma	Liver cancer (Hepatocellular carcinoma)	*TERT* promoter mutations in the analysed series suggested a relationship between these promoter mutations and Wnt pathway gene alterations, including *CTNNB1* in viral-related and non-viral HCCs	The study analysed *TERT* and *CTNNB1* mutations in hepatocellular nodules and suggested further investigation of the mutations on a larger sample
25.	Lee, S.W. et al. 2021	Differences in somatic mutation profiles between Korean gastric cancer and gastric adenoma patients	Gastric Cancer & Gastric Adenoma	There were 31 somatic mutations and 6 frameshift mutations in the analysed cases. GC had more mutated genes and higher allele frequency than GA. *TP53, PIK3CA, ERBB2,* and *RNF43* were identified in 50% of the GC cases	This study analysed somatic mutations and allele frequencies in GC and GA to explain GC molecular pathogenesis and malignant progression
26.	Li, M. et al., 2014	Whole-exome and targeted gene sequencing of gallbladder carcinoma identifies recurrent mutations in the ErbB pathway	Gallbladder Cancer	TP53 had the highest mutation frequency (47.1%), while *KRAS* and *ERBB3* had 7.8% and 11.8% frequency, respectively. ErbB signalling was the most widely mutated pathway. These mutations affected the signalling of *ERBB2*, *ERBB3*, *ERBB4*, and *EGFR*	The study analysed *KRAS*, *TP53*, and *ERBB3* mutations and ErbB signalling and determined that ErbB pathway mutations have a worse outcome
27.	Li, R. et al., 2020	Heterogeneous genomic aberrations in oesophageal squamous cell carcinoma: a review	Oesophageal Cancer	Significant recurrent alterations reported in oesophageal squamous cell carcinoma (ESCC), which is the major oesophageal Cancer (EC) subtype, *were PIK3CA, TP53, NOTCH1, ZNF750, CDKN2A, MLL2, NFE2L2, RB1, FBXW7* and *FAT1*. There was *CDKN2A* deletion and *CCDN1* and *MYC* amplification	This review analysed *TP53* cell cycle regulation and determined that p53, which is the protein product, plays a crucial role in maintaining genomic stability, DNA repair, apoptosis, and cell cycle regulation
28.	Li, W. et al., 2020	Prevalence and characteristics of *PIK3CA* mutation in mismatch repair-deficient colorectal cancer	Colorectal Cancer	*PIK3CA* mutation was common in dMMR tumours. *PIK3CA* coexistence incidence with *KRAS/BRAF/NRAS* was low in the analysed cases. However, the coexistence incidence was higher in *PTCH1* and *HER2* mutations in MMR-deficient tumours than in MMR-proficient tumours	The study discussed *PIK3CA* mutation and determined that MMR status and *PIK3CA* mutation can determine specific CRC groups
29.	Liccardo, R. et al. 2022	Significance of rare variants in genes involved in the pathogenesis of Lynch syndrome	Lynch Syndrome	Deleterious variants were detected in 5.6% of the cases, and unclassified variants were reported in 80.3% of the probands	The study discussed deleterious mutation in the mismatch repair (MMR) pathway and suggested analysis of other MMR genes in LS molecular screening panels
30.	Lin, D. et al., 2017	Genomic and epigenomic heterogeneity of hepatocellular carcinoma intratumoral heterogeneity of hepatocellular carcinoma	Liver Cancer (Hepatocellular carcinoma)	Twenty-nine percent of the driver mutations in the analysed cases were heterogeneous. These mutations had spatial genomic diversity, including *STAG2, TERT, NOTCH2*, and *ARID1A*	The study evaluated HCC biomarkers, including *STAG2*, *TERT*, *NOTCH2*, and *ARID1A*, and suggested further investigations on epigenetic diversity relevant to HCC biology
31.	Liu, G. et al., 2019	Comprehensive multi-omics analysis identified core molecular processes in esophageal cancer and revealed *GNGT2* as a potential prognostic marker	Oesophageal Cancer	Fourteen gene interaction modules were identified; they facilitated gastric acid secretion, binding of the insulin-like growth factor receptor, and protein transport regulation. The module genes we also involved in the pathway signalling of p53 and epidermal growth. Differentially expressed genes *SH3GL2* and *SST* showed sturdy methylation regulation	The study identified *SST* and *SH3GL2* methylation regulation capability and revealed potential regulatory factors in oesophageal cancer development and progression
32.	Lu, J. et al., 2022	Clinicopathological and molecular characteristics of the alpha‐fetoprotein–producing gastric cancer: emphasis on two major subtypes	Gastric Cancer (Alpha-fetoprotein–producing gastric cancer (AFPGC))	TP53 was the highest mutated gene, with an 88.6% mutation rate. Others were CCNEI, MYC, and MCLI in the AFPGC. In the control sample (CGA), *TP5* was at 47.3%. Other mutations included *ARID1A*, *CDKN2A* & *PIK3CA*	The study compared *ZNF217* mutations and *ZNF217* wild-type and established a possible link between cancer prognosis and mutations in *RPTOR, ZNF217, ERBB4,* and *TOP2A*
33.	Luchini, C. et al. 2022	Microsatellite instability in pancreatic and ampullary carcinomas: histology, molecular pathology, and clinical implications	Pancreatic Cancer	Only 1–2% of pancreatic duct carcinoma show MSI. However, 18% of ampullary carcinomas harbour MSI/dMMR alteration, and pancreatic acinar carcinomas harbour 14% molecular alteration	This study evaluated MSI and dMMR and determined the high value of MSI in the clinical management of periampullary tumours
34.	Luchini, C. et al. 2021	Comprehensive characterisation of pancreatic ductal adenocarcinoma with microsatellite instability: histology, molecular pathology, and clinical implications	Pancreatic Cancer	A low prevalence of MSI/dMMR in PDAC, between 1 and 2% was reported. A comparison of conventional PDAC and MSI/dMMR PDAC showed a strong link between MSI/dMMR PDAC with colloid histology. The comparison also revealed a strong relationship with *KRAS/TP53* wild-type molecular background	This systematic review discussed MSI/dMMR PDAC and established a link between the disease and *JAK* gene mutations and *KRAS/TP53* mutations
35.	Ma, H. et al., 2020	Identification of germline and somatic mutations in pancreatic adenosquamous carcinoma using whole exome sequencing	Pancreatic Cancer	Germline mutations identified in *MAP3K1, BCR, PDE4DIP, ALK, AR, USP6, HL-A, SPEN, KMT2D, ZFHX3, NUTM2B* and *MN1*. Somatic mutations were reported in *HRNR, OBSCN, TP53,* and *KRAS.* Additionally, there was a significant link between germline mutations in *USP6* and lymphatic metastasis	This study analysed germline and metastatic mutations in *PDE4DIP, MAP3K1,* and *BCR* genes and suggested the translation of these biomarkers in clinical practice
36.	Machlowska, J. et al. 2020	High-throughput sequencing of gastric cancer patients: unravelling genetic predispositions towards an early-onset subtype	Gastric Cancer	There were seven candidate genes identified in the analysis, including *KIT, STK11, SLX4, RET, MEN1, WRN,* and *FANCM*. Further, 10% of the most deleterious substitutions, including were present in rs1800862, rs10138997, rs2230009, and rs2959656 were present in *RET, FANCM, WRN,* and *MEN1*, respectively	The study discussed seven candidate genes in GC, including *KIT, STK11, SLX4, RET, MEN1, WRN,* and *FANCM*, and suggested further research on GC genetic background
37.	Montal, R. et al. 2020	Molecular classification and therapeutic targets in extrahepatic cholangiocarcinoma	Bile Duct Cancer	The most prevalent mutations reported in the analysis include *KRAS* at 36.7%, *TP53* at 34.7%, *ARID1A* at 14%, and *SMAD4* with 10.7% prevalence	The study analysed *KRAS, TP53, ARID1A,* and *SMAD4* mutations and recommended integrative molecular characterization of distinct eCCA subclasses to explore new therapeutic approaches and study patient stratification
38.	Muller, M. et al. 2020	Genomic and molecular alterations in human inflammatory bowel disease-associated colorectal cancer	Colorectal Cancer (Bowel-related CRC)	CIN was the key feature that caused CRC in inflammatory bowel disease (IBD) cases, occurring in 80% of the cases	The review revealed recurrent *WNT*, *PI13K*, *RTK/RAS*, and *TGFϐ* mutations and suggested further research to understand molecular alterations and pathogenesis leading to CRC related to human-inflammatory bowel disease
39.	Rodriguez, M. et al. 2016	Gastrointestinal stromal tumors (GISTs) and second malignancies: a novel “sentinel tumor”? A monoinstitutional, STROBE-compliant observational analysis	Gastric Cancer	More than half (52%) of the second tumours in the study originated from the gastrointestinal tract. Furthermore, 35.9% of GIST cases had a second neoplasm. There was a wild-type *KRAS* mutation in all the analysed cases	The study evaluated molecular analysis in *PDGFR-α* and *KIT* genes and suggested further follow-up on research on the molecular mechanism of the unknown common molecular mechanism in second secondary tumours
40.	Scarpa, A. et al. 2017	Whole-genome landscape of pancreatic neuroendocrine tumours	Pancreatic Cancer (PanNETs)	Mutations in *BRACA2, MUTYH,* and *CHEK2* occurred in 17% of the cases. Gene fusions and point mutations were found in mTOR signalling activation, DNA damage repair, and telomere maintenance	The study described *MEN1* and *VHL* mutations in PanNETs and mTOR signalling and suggested identified subgroup tumours associated with HIF signalling and hypoxia
41.	Setia, N. et al. 2021	Morphologic and molecular analysis of early‐onset gastric cancer	Gastric Cancer	A high *CDH1* mutation rate was reported in 22.2% of the early onset GC cases and in 11.4% of the traditional GC cases. Additionally, the *TP53* gene revealed regular transition mutations (65.5%)	This study explored *CDH1* and *MUTYH* mutations and determined that endogenous factors that cause germline alterations and cytosine deamination may be associated with cancer pathogenesis in cancer-susceptible genes
42.	Shimizu, T. et al. 2014	Accumulation of somatic mutations in *TP53* in gastric epithelium with Helicobacter pylori infection	Gastric Cancer	Analysis revealed *TP53* mutations in 44.1% of the cases and *ARID1A* mutations in 14.7% of the cases	The study discussed *TP53* and *ARID1A* mutation in GC and determined that various genes in the gastric mucosal tissues can mutate when there is H pylori infection
43.	Siraj, T. et al. 2018	*MED12* is recurrently mutated in Middle Eastern colorectal cancer	Colorectal Cancer	The study found an inverse correlation between differentially expressed *MED12* and transforming growth factor TFG- ϐ signalling	The study examined the potential role of *MED12* mutations in CRC, determined that *MED12* recurrently mutates, and proposed the findings corroborated previous proposals of the mutation’s role
44.	Tan, M. et al., 2021	Whole genome sequencing identifies rare germline variants enriched in cancer related genes in first-degree relatives of familial pancreatic cancer patients	Pancreatic Cancer	*PALD1* showed the highest statistical significance, and *LAMB4* was the most expressed ECM protein in the pathway	The study discussed rare germline PTVs in familial pancreatic cancer and suggested that the identified variants could be useful in predicting risk among high-risk individuals predisposed to this cancer
45.	Tran, T. et al., 2013	Molecular changes in the phosphatidylinositide 3‐kinase (PI3K) pathway are common in gastric cancer	Gastric Cancer	P13 pathway alterations were detected in 54% of the gastric tumours. *PIK3CA* mutations were detected in 4.9% of the tumours and 13.1% of the copy number gain	The study screened *PIK3CA* and copy number gain in GC tumours and established that *P13K* alterations were common in GC tumours
46.	Van den Broek, E. et al. 2016	Genomic profiling of stage II and III colon cancers reveals *APC* mutations to be associated with survival in stage III colon cancer patients	Colon Cancer	Frequent chromosome loss of 18q12.1-18q12.2 was identified in relapsed tumours. *APC* and *TP53* mutations were associated with somatic copy number aberrations (CNAs)	The study analysed the mutation status of *KRAS, APC, PIK3CA, SMAD4*, *FBXW7, BRAF,* *TP53,* and *NRAS* and concluded that the strongest association between genomic alterations and clinical outcome was in *APC*
47.	Wang, X. et al. 2023	An exploration of gastric cancer with heterogeneous mismatch repair status	Gastric Cancer	Heterogenous MMR protein was reported in twelve GC cases. Furthermore, *TP53* mutation accompanied *MLH1* heterogenous staining	The study evaluated four key proteins, including *MSH2*, *MSH6*, *MLH1*, and *PMS2*, and proposed that MMR heterogeneity and microsatellite status be considered in GC pathological diagnosis process
48.	Xue, R. et al. 2016	Variable intra-tumor genomic heterogeneity of multiple lesions in patients with hepatocellular carcinoma	Liver Cancer	All lesions had ubiquitous mutations that varied among the patients; the least mutation percentage was 8%, while the highest was 97%. Satellite nodules shared 90% of the mutations identified in the original lesions	The study identified genomic abnormalities, including copy number variations and somatic mutations, and suggested further genomic comparison to provide valuable information on genomic changes in HCC
49.	Yan, Z. et al., 2016	Comparative study of mutations in SNP loci of K*RAS*, *hMLH1*, and *hMSH2* genes in neoplastic intestinal polyps and colorectal cancer	Colorectal Cancer	The SNPs mutation rate in codons 12 and 13 of *KRAS* and *hMSH2* was lower in the observation group than in the control group. The SNP mutation rate at the *hMLH1* gene was meaningfully higher in the observation group than in the control group	The study discussed mutation in *KRAS, hMLH1*, and *hMSH2* and established a clinical relevance and significance between CRC and neoplastic intestinal polyps
50.	Yoshida, T. 2019	Identification of early genetic changes in well-differentiated intramucosal gastric carcinoma by target deep sequencing	Gastric Cancer	*TP53* mutation was 54.8%, and Wnt signalling pathway genes mutation in *APC* and *CTNNB1* genes was 19.4% of the cases. Macroscopic protrusion and H. Pylori infection were linked to these mutations	The study discussed *TP53*, *APC*, and *CTNNB1* mutations and suggested further investigation of determined that Wnt-signalling and *TP53* were frequent and mutually exclusive in the early stages of GC
51.	Gao, Y.X. et al. 2025	Whole-Genome and RNA Sequencing Reveal Novel Insights into the Pathogenesis of Colorectal Cancer in Persons Living with HIV	Colorectal Cancer	Whole-genome and RNA sequencing identified 1,705 differentially expressed genes, including interferon-stimulated genes such as *IFI44*, *MX1, OAS1, OAS3, BST2,* and *IFIT1,* together with SNP and indel alterations. Immune infiltration differences were also reported	Whole-genome and RNA sequencing identified interferon-stimulated genes including *IFI44, MX1, OAS1,* and *OAS3* as key regulators. These findings indicate that immune-related pathways contribute to colorectal carcinogenesis in immunocompromised populations
52.	Jurecka-Lubieniecka, B. et al. 2025	Germline Mutations in DNA Repair Genes in Patients with Pancreatic Neuroendocrine Neoplasms: Diagnostic and Therapeutic Implications	Pancreatic Cancer (Neuroendocrine tumours)	Germline mutations in DNA repair genes, including *BRCA1, BRCA2, CHEK2, MLH1, MSH2, MSH6,* and *PMS2*, were identified, implicating both homologous recombination and mismatch repair pathways in tumour susceptibility	Germline mutations in DNA repair genes including *BRCA2, CHEK2, ATM,* and *MSH2* were identified in pancreatic neuroendocrine neoplasms. These findings suggest that defective DNA repair mechanisms contribute to tumour susceptibility and progression
53.	Kumar, S. et al. 2025	Next-Generation Sequencing Identifies Novel Germline Mutations in Patients with Budd-Chiari Syndrome-Associated Hepatocellular Carcinoma	Liver Cancer (Hepatocellular carcinoma)	Whole exome sequencing identified 1849 significant mutations across 305 genes, including frequent mutations in mucin family genes such as *MUC3A, MUC4, MUC6,* and *MUC16.* Gene ontology analysis implicated extracellular matrix and glycosylation-related pathways	Whole-exome sequencing identified mutations in mucin family genes including *MUC3A, MUC4, MUC6,* and *MUC16*. These findings suggest a role for extracellular matrix and glycosylation pathway alterations in hepatocellular carcinoma pathogenesis
54.	Lin, M.T. et al. 2025	Highly sensitive and specific markers for detection of mismatch repair deficiency by next-generation sequencing	Colorectal Cancer	Mismatch repair deficiency (dMMR) was associated with a significantly higher burden of insertion/deletion (indel) mutations within exonic homopolymers. These mutations predominantly occurred in longer nucleotide repeats and were enriched at specific hotspot loci	This study identified mismatch repair deficiency through exonic homopolymer mutations and indel signatures, highlighting *MLH1, MSH2, MSH6,* and *PMS2* involvement. These findings support the role of defective DNA mismatch repair in driving genomic instability in colorectal carcinogenesis
55.	Skerl, P. et al. 2025	Real-World Evaluation of Microsatellite Instability Detection via Targeted NGS Panels in Routine Molecular Diagnostics	Colorectal Cancer	Microsatellite instability assessment using targeted NGS involved mismatch repair-related markers including *MLH1, MSH2, MSH6, PMS2*, together with tumour mutational burden metrics. The study reported clear separation of MSI-H and MSI-S groups, although colorectal cancers showed broader MSI score variability	Microsatellite instability was detected using targeted NGS through mutations in *MLH1, MSH2, MSH6,* and *PMS2*. This reinforces the role of mismatch repair deficiency as a key mechanism driving colorectal cancer development
56.	Tanabe, H. et al. 2025	Early genetic events in the colorectal carcinogenic pathway of familial adenomatous polyposis and sporadic polyp: germline and somatic alterations in carcinogenesis	Colorectal Cancer	Early colorectal tumorigenesis was characterised by predominant *APC* alterations, with additional mutations in *KRAS, FBXW7, CTNNB1, BRAF,* and *PIK3CA*. The findings supported *APC*-driven early carcinogenic events in both familial adenomatous polyposis and sporadic lesions	Early colorectal tumorigenesis was driven by *APC* mutations, followed by *KRAS, CTNNB1,* and *FBXW7* alterations. These findings support a stepwise mutation model in colorectal carcinogenesis involving progressive accumulation of oncogenic events
57.	Xan, L. et al. 2025	Genomic landscape and potential therapeutic targets in alpha-fetoprotein-producing gastric cancer	Gastric Cancer	Frequent mutations were identified in *TP53*, with additional alterations in *ERBB2, EGFR, CCNE1, ARID1A,* and *LRP1B*, supporting the involvement of receptor tyrosine kinase and cell-cycle related pathways in alpha-fetoprotein-producing gastric cancer	Frequent mutations in *TP53, ERBB2, EGFR, CCNE1,* and *ARID1A* were identified in alpha-fetoprotein-producing gastric cancer. These alterations indicate enhanced proliferative signalling and genomic instability underlying this aggressive gastric cancer subtype

For synthesis purposes, findings were categorized by cancer type, specifically CRC, gastric, liver, pancreatic, and oesophageal cancers, along with other DSCs such as gallbladder cancer (GBC) and Lynch syndrome (Table 2).

### Molecular pathogenesis by cancer type

*Colorectal cancer (CRC)* was the most frequently studied subtype [16–26]. Common mutations were identified in *PIK3CA*, *KRAS*, *MED12*, *FGFR*, *BRAF*, *NRAS*, and *IDH1/2* [9, 10, 20, 21, 23, 25]. Some studies reported variability in *KRAS* mutation frequency and a higher proportion of *hMLH1* mutations in CRC patients [16, 23, 27]. In addition to mismatch repair deficiency, mutations in the *DNA polymerase epsilon* (*POLE*) gene have been identified as an alternative mechanism driving hypermutation in colorectal cancer [63, 64] *POLE*-mutated tumours are characterised by an ultra-mutated phenotype and may exhibit increased immunogenicity, similar to MSI-high tumours. Although less frequently reported, these mutations have important implications for tumour classification and potential responsiveness to immunotherapy, highlighting the need for their inclusion in molecular profiling frameworks. Nigerian CRC cases showed higher microsatellite instability (MSI) and fewer *APC* and Wnt pathway mutations, but more frequent alterations in the RAS pathway [28, 65]. Bioinformatics analyses further highlighted hub genes such as *WNT2*, *SFRP2*, *ZNF726*, and *BMP2* [22]. *KRAS*-mutated metastatic CRC was associated with elevated protein expression levels [20]. Subtype-specific patterns were also noted, including distinct mutational landscapes in inflammatory bowel disease-associated CRC, rectal squamous cell carcinoma, anal squamous cell carcinoma, and rectal adenocarcinoma (rADC) [26, 29, 30, 66]. One study proposed a CRC progression model beginning with mismatch repair (MMR) deficiency, followed by mutations in *KRAS* and *APC* [27, 67]. In addition, emerging evidence suggests that clinicopathological factors such as tumour sidedness and sex may influence the molecular landscape of colorectal cancer. Right-sided and left-sided CRCs have been shown to differ in their mutational profiles, with right-sided tumours more frequently associated with microsatellite instability, *BRAF* mutations, and CpG island methylator phenotype, whereas left-sided tumours more commonly harbour chromosomal instability and *KRAS* mutations [68]. Sex-based differences have also been reported, with variations in mutation frequency, immune response, and survival outcomes between male and female patients [69]. These factors highlight the importance of incorporating clinical and demographic stratification in the interpretation of molecular data.

*Gastric cancers* were addressed in studies that explored diverse molecular features, including classifications based on The Cancer Genome Atlas (TCGA), PI3K pathway alterations (present in 54% of cases), and variable MMR status [31–33]. Early gastric cancers showed frequent mutations in *TP53*, *APC*, and *CTNNB1*, often involving the Wnt signaling pathway. Subtype differences were also documented. Alpha-fetoprotein-producing gastric cancer demonstrated higher lymphovascular invasion compared to common gastric adenocarcinoma, while early-onset cases were enriched for *CDH1* mutations [34, 35]. This supports the classification of AFPGC as a distinct molecular subtype characterised by enhanced proliferative signalling and genomic instability. Additional studies reported novel mutations in *ESR1* and *EZR1* in familial intestinal-type gastric cancer, frameshift mutations in *ROBO1* and *ROBO2* in MSI-high tumours, and highlighted the rarity of somatic *SPOP* mutations, despite their protein overexpression across cancers [36–38]. Alterations in receptor tyrosine kinases, particularly *ERBB2* and *EGFR*, were also frequently observed, supporting their role in driving tumour proliferation and progression through activation of downstream signalling pathways such as PI3K/AKT and MAPK [70]. Other studies focused on mutation burdens in gastric adenomas versus carcinomas, and increased mutation rates in secondary gastrointestinal stromal tumours [39, 40].

*Liver cancer* particularly hepatocellular carcinoma (HCC), was characterized by frequent alterations in cancer stem cell-related pathways and common driver mutations in *TERT* and *CTNNB1*. Multi-nodular HCC cases demonstrated shared mutational clonality across tumour sites [41]. Intrahepatic cholangiocarcinoma (iCCA) was associated with chronic biliary inflammation and a distinct mutational spectrum. Studies focusing on hepatitis B virus related HCC reported considerable heterogeneity, with mutations in *ARID1A*, *STAG2*, *NOTCH2*, and *TERT*, alongside widespread *TP53* mutations across tumour regions [42, 43]. Recent genomic studies in Budd–Chiari syndrome-associated HCC have further demonstrated novel germline mutations, highlighting additional molecular heterogeneity within HCC subtypes [71]. Fibrolamellar carcinoma (FLC) was defined by recurrent *DNAJB1–PRKACa* fusions and *TERT* promoter mutations [44]. In cholangiocarcinoma, common mutations included *KRAS*, *TP53*, *ARID1A*, and *SMAD4*, with one-quarter of cases harbouring targetable alterations [45].

*Pancreatic cancer* demonstrated low MSI prevalence (1–2%) in pancreatic ductal adenocarcinoma (PDAC) [46]. Germline mutations were identified in *PALD1*, *COL4A2*, *LRP1B*, *AHDC1*, *CYLC2*, *ZFYVE9*, and *BRD3* in a Danish cohort, and in *BRCA2*, *MUTYH*, and *CHEK2* in pancreatic neuroendocrine tumors (PanNETs) [47–49]. Adenosquamous carcinoma cases showed co-occurrence of germline mutations (e.g., *MAP3K1*, *BCR*, *ALK*) and somatic mutations in *TP53* and *KRAS*. PanNETs further exhibited frequent mutations in *TP53*, *KRAS*, and *RB1*, implicating chromatin remodeling and DNA repair pathways, along with mTOR signaling [50]. Although uncommon, *POLE* mutations have been reported in pancreatic cancers and may contribute to hypermutated phenotypes in a subset of cases [72, 73]. Furthermore, sex-specific differences in PDAC have been increasingly recognised, with studies reporting variations in tumour biology, immune microenvironment, and clinical outcomes between male and female patients [47, 74]. These differences may influence the prevalence and functional impact of key mutations, including *KRAS* and DNA damage repair genes, and warrant further investigation in future molecular studies of PDAC [47, 74].

*Oesophageal cancer* particularly oesophageal squamous cell carcinoma, was noted for its high prevalence of structural variants, single nucleotide variants, and copy number alterations [51]. Frequent *TP53* mutations were observed, contributing to genomic instability and dysregulation of cell cycle control. Transcriptomic analyses identified over 7,000 differentially expressed genes and multiple gene interaction modules implicated in disease progression [52].

*Other DSCs* included GBC, where *TP53* (47.1%), *KRAS* (7.8%), and *ERBB3* (11.8%) mutations were most frequently reported, with the ErbB signaling pathway altered in 36.8% of cases [53]. In Lynch syndrome, rare pathogenic variants were found in 5.6% of individuals, and while 80.3% of variants were categorized as variants of uncertain significance (VUS), many were deemed clinically relevant [54]. These findings further emphasise the role of receptor tyrosine kinase signalling and mismatch repair deficiency in less common digestive system cancers.

To enhance clarity and facilitate interpretation of complex molecular data, key findings have been summarised using structured tables and visual representations. These include an overview of study characteristics (Table 1), tabulated summaries of included studies (Table 2), and a synthesis of molecular pathogenesis across DSC subtypes (Table 3). In addition, a qualitative visual comparison of recurrently reported genes and pathways across DSCs is presented in Fig. 2. Such visualisation approaches improve readability and allow for clearer comparison of molecular alterations across cancer types [11, 13].

**Table 3:** Molecular pathogenesis of DSCs analysed in this scoping review

#	Types of digestive system cancer	Gene(s) involved	Biological function/ pathway involved	Genetic variance resulting in the pathogenesis of cancer	Identified gap(s) from this scoping review
1.	Colorectal cancer (CRC)	*KRAS, TP53, BRAF, APC, FBXW7, CTNNB1, PIK3CA, MLH1, MSH2, MSH6, PMS2, IFI44, MX1, OAS1, OAS3*	Lynch syndrome (LS) CRC arises through three different pathways. CRC in LS can originate from mismatch repair (MMR)-proficient adenomas when there is a secondary inactivation in the MMR system. MMR deficiency is the possible initiating event that leads to CRC development in LS-CRC. *KRAS* and *APC* mutations occur after the MMR deficiency onset—tumours without polypus growth evidence present with *CTNNB1* and *TP53* mutations The mutational profile in rSCC is *PIK3CA*, *PTEN*, *TP53*, *ATM*, *BCL6*, *SOX2*. The most frequent mutated genes in HPV-related rSCC are *PIK3CA* and *PTEN*. Notably, *PIK3CA* is also relatively mutated in other cancers associated with HPV (human papillomavirus). Therefore, PI3K/Akt/mTOR pathway plays a significant role in rSCC carcinogenesis CRC is propelled by various driver gene alterations that start after disease initiation and continue accumulating with CRC progression. Additionally, MMR deficiency contributes to genomic instability through the accumulation of insertion–deletion mutations within exonic homopolymer regions. Emerging evidence indicates that interferon-stimulated gene signalling and immune-related pathways contribute to colorectal carcinogenesis, particularly in immunocompromised populations The Wnt signaling pathway is a fundamental pathway in CRC pathogenesis. Abnormal canonical Wnt signaling is a notable occurrence in CRC carcinogenesis, characterised by irregular activation of the pathway	MSI status in hereditary CRCs is linked to pathogenic variants in MMR genes The first alteration in sporadic CRCs is a hypermutated group of defective DNA with *POLE* mutations and microsatellite instability (MSI). The second group is the non-hypermutated group containing various somatic copy number alterations involving oncogenic activation of *PIK3CA* and *KRAS* mutations and loss of tumor suppressor genes *APC* and *TP53* Somatic mutations in Rectal adenocarcinoma and colonic adenocarcinoma tumours indicate that both diseases follow similar molecular pathogenesis. Colorectal adenocarcinoma starts with *APC* inactivation at 5q21. Consequently, the sequence leads to *KRAS* and *TP53* mutations and heterozygosity loss RPPL p.K15fs hotspot mutation found in IBD-CRCs is also present in colorectal, endometrial, and gastric cancers. Somatic copy number alterations (SCNAs) in IBD-CRCs are more prevalent non- hypermutator cases than in hypermutator cases (Din, 2018) High c-MET expression in *KRAS* mutation in mCRC compared to *KRAS* mutation in CRC shows c-MET inhibitors MSI is one of the molecular events in GC progression. Chromosomal instability is the most predominant genomic instability in GI tract cancers. Furthermore, dMMR colorectal cancers exhibit recurrent insertion–deletion mutations within exonic homopolymer regions, reflecting defective DNA mismatch repair mechanisms. Early colorectal carcinogenesis is initiated by *APC* mutations, followed by alterations in *KRAS*, *CTNNB1*, *FBXW7*, and *PIK3CA*	The role played by mutations in MMR genes in the progression of CRC carcinogenesis in LS requires further research Molecular alterations that cause IBD-CRC (inflammatory bowel disease-associated CRC) require further analysis Analysis of distinct CRC subtypes to determine their unique molecular basis and determine similarities/differences in the somatic mutations is essential. CRC tumours originate from different tissues with unique clinical features, but these tumours also share several features. The interaction between immune dysregulation and genomic alterations in colorectal cancer, particularly in immunocompromised populations, remains incompletely understood
2.	Gastric cancer	*PIK3CA, BCOR, ARID1A, ERBB2, EGFR, CCNE1, LRP1B*	The driving mutations in *FIGC* are *ESR1*, *EZR*, and *IGF2R* are biomarkers that play hub roles in the disease pathogenesis The *EZR*, *IGF2R*, and *ESR1* protein structure changes significantly after mutation. The genetic mutations alter the protein structure, impede protein function, and further alter the phenotype. Changes in the *ESR1*, *EZR*, and *IGF2R* affect the signal transduction of the MAPK/ERK signaling pathway (Chen et al. 2018) Gastric carcinoma with lymphoid stroma is a rare variant characterized by MMR (mismatch repair) deficiency and EBV (Epstein-Barr virus) positivity. Alterations occur in *KRAS*, *PIK3CA*, *ARID1A*, and *TP53* genes Similar to CRC, a somatic frameshift mutation alters *ROBO1* and *ROBO2*, which are axon guidance genes, in GC. The mutation alters *ROBO2* genes unrelatedly to MSI status. Additionally, receptor tyrosine kinase signalling and cell-cycle regulatory pathways contribute to tumour proliferation and progression in specific gastric cancer subtypes	Key genetic alterations in EBV-associated GC include *PIK3CA*, *BCOR*, and *ARID1A* mutations and PD-L1/2 amplifications. DNA methylation involves *CDKN2A* silencing and *MLH1* absence. Genomically stable tumours are characterised by *CLDN18–ARHGAP26* fusion and mutations in *RHOA* and *CDH1* genes, along with CpG methylation patterns In addition, alpha-fetoprotein-producing gastric cancer (AFPGC) represents a distinct molecular subtype characterised by a high mutational burden involving *POLE*, *TP53*, *MYC*, *PKHD1*, and *CCNE1*. Additional alterations in *ERBB2*, *EGFR*, *ARID1A*, and *LRP1B* further indicating enhanced proliferative signalling and genomic instability. In contrast, mutations in *JAK2*, *CDKN2A*, *PTEN*, and *SMAD4* are more commonly observed in conventional gastric adenocarcinoma but are typically absent in *AFPGC*	The causal relationship between *TP53* mutation accumulation and *AID* activity in gastric cancer pathogenesis is not well defined Heterogenous mismatch repair and microsatellite status in GC need to be assessed to determine the relationship between gene mutations and the staining of MMR proteins The molecular mechanisms underlying the aggressive clinical behaviour of AFPGC remain to be fully elucidated
3.	Liver cancer	*KRAS, BRAF, EGFR, ARID1A, TP53, MUC3A, MUC4, MUC6, MUC16*	Loss of p53 and chronic biliary inflammation reveals the role of the biliary epithelium as the source Key signalling pathways in ICC pathogenesis are the presence of epidermal growth receptor *ERBB2*, *EGFR*, and *RAS*, increased P13K/AKT/mTOR, and hepatocyte growth factor JAK/STAT Common activating mutations in *KRAS*, *BRAF*, *EGFR*, and *IDH1/IDH2* are reported in iCCA. There are inactivating mutations in *TP53*, but *PIK3CA* and *NRAS* are rare in iCCA. The most affected receptor is the ErbB-family. Additionally, alterations in mucin genes may influence extracellular matrix organisation and glycosylation processes, contributing to tumour development The most prevalent mutated genes in bile duct cancer include *KRAS*, *ARID1A*, and *TP53*	iCCA pathogenesis is associated with chronic biliary inflammation that leads to bile duct damage and obstruction. Dysregulation of key signalling pathways, including PI3K/AKT/mTOR and RAS/RAF/MAPK, is a distinct feature of iCCA. Common activating mutations in *KRAS*, *BRAF*, *EGFR*, and *IDH1/2* have been reported, while inactivating mutations in *TP53*, *PIK3CA*, and *NRAS* are less frequent Fibrolamellar carcinoma is characterised by the *DNAJB1–PRKACA* fusion transcript, along with mutations in genes such as *CTNNB1*, *SF3B1*, and *ZFHX3*. Mutations in *CTNNB1* are commonly associated with hepatocellular carcinoma, whereas *TP53* mutations are strongly linked to hepatitis B virus infection Additional mutations involving genes such as *MLL*, *MLL3*, *MLL4*, *AXIN1*, *NFE2L2*, and *CDKN2A* contribute to hepatocarcinogenesis by affecting Wnt signalling, DNA repair, chromatin modification, and oxidative stress pathways Recent genomic studies indicate that alterations in mucin family genes, including *MUC3A*, *MUC4*, *MUC6*, and *MUC16*, may contribute to tumour development through extracellular matrix remodelling and glycosylation processes In bile duct cancer, recurrent chromosomal alterations have been identified in *CCNE1*, *ERBB2, MDM2, YEATS4*, and *CDK4*, converging into RTK-RAS-PI3K, TP53-RB, and *TGF-β* signalling pathways. These alterations define distinct tumour subtypes, including proliferative, metabolic, immune, and mesenchymal classes	Molecular alterations and pathways involved in most liver cancer subtypes have been discussed, including iCCA, bile duct cancer, and HCC. The molecular landscape of FLC has been discussed but discussed in the studies. However, the information on FLC pathways is inadequate. The functional significance of mucin gene alterations in hepatocellular carcinoma remains unclear and requires further investigation
4.	Pancreatic cancer	*TP53, KRAS, BRCA2, CHEK2, ATM, MSH2, PMS2, EPCAM, MUC1, MUC4, MUC16*	Gene mutation in PanNETs mostly affects the *TP53* gene. These tumours also cause RB1 expression loss. Most carcinomas have singular mutations in *ARID1A*, *TP53*, *CDKN2A*, and *LRPIB* MSI/dMMR in PDAC reveals a link between molecular alteration and *KRAS/TP53*-wildtype mutation. Most of the PDAC cases harbour these mutations. Therefore, MSI/dMMR is a unique entity in PDAC categorization PanNETs molecular pathology involves mTOR signalling activation that is triggered by new gene fusion events PanNET tumours with a *MUTYH* signature carry a germline inactivation mutation that concurrently results in wild-type allele loss Emerging evidence highlights the contribution of germline DNA repair gene alterations, including *BRCA2*, *CHEK2*, and *ATM*, to pancreatic tumour susceptibility and progression. Additionally, mucin-related pathways may contribute to tumour biology through altered cell adhesion and extracellular interactions	In PanNETs, the distribution of protein-to-protein interaction links occurs in the signalling pathways. Therefore, the disease causes genetic changes in the SMAD2/3 signalling pathway by altering its regulation. *ESR1* and *GATA3* mutated genes regulate SMAD2/3 signalling. *KMT2D* mutated gene is involved in mTOR signalling and histone methylation. *KDR*, *PDGFRA*, and *FGRFR2* alter RAS angiogenesis and signalling Notably, *MUTYH* deficiency plays a crucial role in PanNET Premature truncating variants (PTV) in pancreatic cancer shorten genes’ protein-coding sequence. Therefore, these variants alter the function of the hosting gene by increasing or decreasing the gene function. Recent studies have identified pathogenic germline variants in DNA repair genes such as *BRCA2*, *CHEK2*, and *ATM* in pancreatic neuroendocrine tumours, supporting a role for inherited susceptibility. Mucin-related alterations may further contribute to tumour progression through effects on cell adhesion and extracellular matrix interactions	Genetic variants have been analysed to determine PDAC risk among relatives of FPC. However, the genetic basis of cancer susceptibility within the PDAC high-risk category is missing Classification of missense and nonsense mutations in PanNETs – candidate genes reveal molecular pathogenesis Germline and somatic mutations in PAC have been identified in one study, but there is inadequate information explaining the relationship between the mutations and *PASC* progression. The contribution of germline DNA repair variants to pancreatic tumour initiation and progression remains insufficiently characterised, particularly in diverse populations. The functional impact of mucin-related alterations in pancreatic carcinogenesis also requires further investigation
5.	Oesophageal cancer	*TP53, SH3GL2, SST, GNGT2*	*TP53*, which is a tumour suppressor gene, is the most mutated gene in ESCC. The protein product p53 regulates the cell cycle, maintains genomic stability, repairs cell DNA, facilitates apoptosis, and regulates cellular senescence. The protein loss mutation leads to cell dysregulation. This process contributes to GI/S transition and is amplified in ESCCs. Recent multi-omics analyses have identified *SH3GL2* and *SST* as key regulators of methylation-related pathways, while *GNGT2* has been implicated in promoting tumour cell proliferation, suggesting additional layers of regulatory complexity in oesophageal carcinogenesis	Detection of abnormal activation of the RTK-MAPK-P13K pathway is common in cancers. *PIK3CA* frequently mutates in cancers, including EC. There is increased kinase activity in H1047 and E542, and E545 exons *SST* gene is a primary gene involved in oesophageal cancer progression regulation. *GNGT2* is capable of promoting oesophageal cancer cell line proliferation. Differential gene expression involving *SH3GL2*, *SST*, and *GNGT2* contributes to tumour progression through epigenetic regulation and signalling pathway activation	ESCC molecular processes and genomic anomalies are explained comprehensively in the studies. However, a fundamental analysis of other mutated genes, excluding *TP53*, which is identified as a tumour suppressor gene in ESCC, is lacking. The integration of genomic and transcriptomic alterations in oesophageal cancer remains limited, and further studies are required to clarify the functional role of newly identified regulatory genes
6.	Gall bladder	*TP53, ERBB3*	The highest mutation frequency in GBC is *TP53*, and the lowest frequency is *KRAS*. Genomic studies have identified recurrent alterations in ERBB family signalling, including *ERBB2* and *ERBB3*, supporting their role in tumour proliferation and progression through downstream pathway activation	ErbB signaling is the most widely mutated pathway in GBC. ErbB receptors play a role in tumour development, while the overexpression of *ERBB2* and *EGFR* has been shown to contribute to the disease progression. Overexpression of *ERRBB2/3* mutant has been linked to a significant proliferation increase in the epithelial cell lines Furthermore, overexpression in either mutant or wild-type *ERBB2* has led to increased HEK293T cell proliferation Silencing of *ERBB2*, *ERBB3*, and *EGFR* inhibits the growth of cell lines in GBC and impairs cell motility. Therefore, ErbB mutants have an oncogenic potential; the activating alterations underpin the ErbB family receptors’ significance in GBC development and progression. Whole-exome sequencing studies have identified recurrent mutations in ERBB pathway genes, including *ERBB2* and *ERBB3*, highlighting their role in gallbladder carcinogenesis and potential as therapeutic targets	The only study available on GBC focused on ErbB receptors. Recurrent mutations in the pathway are discussed comprehensively, but there is inadequate information on the entire spectrum of somatic mutations in GBC. Despite advances in identifying ERBB pathway alterations, the broader genomic landscape of gallbladder cancer remains incompletely characterised, requiring larger studies to define clinically actionable targets

**Fig. 2:**
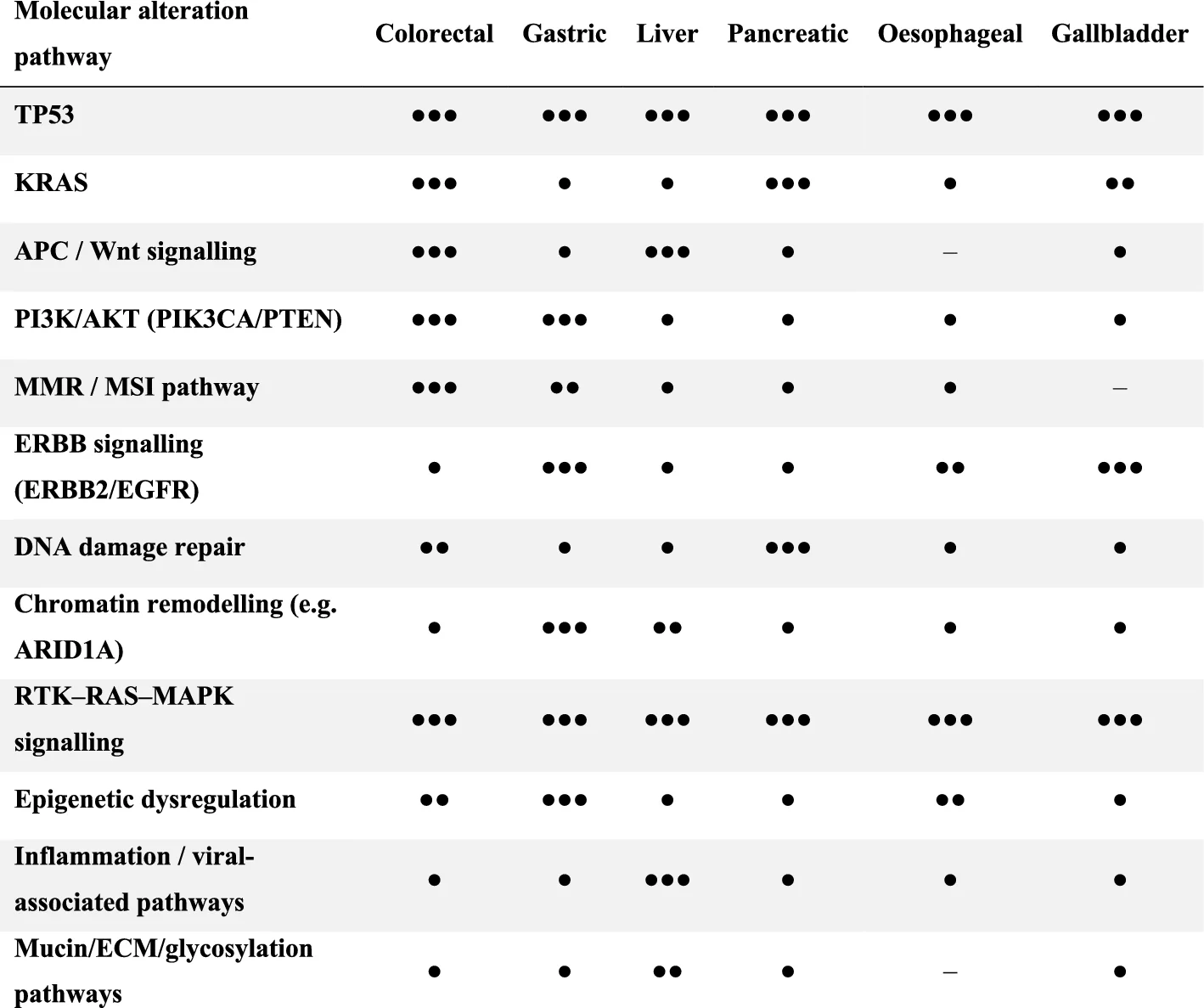
Comparative overview of recurrent molecular alterations and pathways across digestive system cancers

## Discussion

This scoping review synthesizes recent evidence on the molecular pathogenesis of DSCs, encompassing genetic, epigenetic, and transcriptomic alterations across a range of gastrointestinal malignancies. Through the identification of recurrent mutations, pathway dysregulations, and emerging biomarkers, the review highlights key molecular features of DSCs while also delineating significant gaps in the literature, particularly in relation to hereditary variants and underrepresented cancer subtypes.

Consistent with previous studies, the findings reaffirm the central role of key oncogenes and tumour suppressor genes such as *TP53*, *PIK3CA*, *CDKN2A*, and *KRAS* across various DSCs. Tables 2 and 3, together with the comparative summary in Fig. 2, demonstrate that *KRAS* mutations remain a shared oncogenic driver across colorectal, pancreatic, and gallbladder cancers, contributing to therapeutic resistance. *TP53*, the most frequently mutated gene across cancers, contributes to genomic instability and loss of apoptosis regulation, particularly in HCC, gastric cancer, and CRC. Similarly, mutations in *PIK3CA* activate the PI3K/AKT/mTOR pathway, promoting cellular proliferation and survival, while alterations in *CDKN2A* disrupt cell cycle checkpoints [26].

Collectively, these findings suggest that while core oncogenic drivers such as *TP53*, *KRAS*, and *PIK3CA* are conserved across DSCs, their functional impact is highly context-dependent, shaped by tumour-specific microenvironments, tissue origin, and co-occurring genomic alterations. To address potential oversimplification arising from pooling diverse tumour entities, we interpreted molecular alterations within both cross-cancer and tumour-specific contexts.

For instance, mutations in *KRAS*, *TP53*, and *PIK3CA* were consistently observed across multiple DSC subtypes, supporting their role as common drivers of tumourigenesis [2, 20]. However, the relative frequency, co-occurrence patterns, and downstream pathway activation of these mutations vary substantially between cancers [20, 21]. Colorectal cancer frequently demonstrates mismatch repair deficiency and microsatellite instability, whereas pancreatic ductal adenocarcinoma is characterised by near-universal *KRAS* mutations with low MSI prevalence [31, 47]. Similarly, hepatocellular carcinoma and cholangiocarcinoma exhibit distinct mutational spectra shaped by chronic inflammatory and viral aetiologies [43, 44]. These findings underscore that, although shared molecular mechanisms exist, DSCs represent a heterogeneous group of malignancies requiring subtype-specific interpretation of molecular alterations.

Importantly, mutations in *KRAS*, a critical mediator of the RAS/RAF/MEK/ERK cascade, were identified across CRC, PDAC, and GBC, reinforcing its role as a shared oncogenic driver in DSCs. These mutations have been linked to constitutive activation of downstream signalling pathways, contributing to resistance to anti-*EGFR* therapies in CRC [1, 55]. Additionally, the frequent involvement of chromatin remodelling genes such as *ARID1A*, along with DNA damage repair genes including *BRCA2* and *CHEK2*, in PanNETs and iCCA, indicates a more diverse and complex genomic landscape [48]. These alterations suggest that beyond canonical cancer pathways, alterations in genome maintenance mechanisms, such as defective DNA repair and dysregulation of gene expression, may also contribute significantly to tumour progression and influence therapeutic responses in DSCs.

Microsatellite instability (MSI), resulting from defects in the MMR system, was predominantly reported in CRC and gastric cancer. MSI-high tumours typically exhibit hypermutated phenotypes, neoantigen generation, and responsiveness to immune checkpoint inhibitors [52]. However, the prevalence of MSI across DSCs remains variable, with low frequency in pancreatic and oesophageal cancers. Given this variability, standardising MSI screening in clinical practice is essential to ensure appropriate identification of patients who may benefit from immunotherapy.

Additionally, epigenetic silencing of *MLH1* via promoter hypermethylation has been implicated in MMR deficiency, particularly in CRC and gastric cancer, where MSI is more frequently observed. In this scoping review, Kim and Seo (2022) reported *MLH1* hypermethylation in Epstein-Barr virus-positive and MSI-high gastric cancer, reinforcing the role of epigenetic inactivation of DNA repair genes [56]. This finding is consistent with broader literature indicating that *MLH1* silencing contributes to the MSI phenotype in CRC and gastric cancer [57]. The presence of such epigenetic changes provides further insight into the interplay between genetic and epigenetic events in tumour evolution and highlights the potential for incorporating methylation-based diagnostics into clinical practice to complement existing MSI testing protocols [58].

In addition to point mutations and copy number variations, epigenetic dysregulation plays a substantial role in DSC pathogenesis. Several studies included in this review explored epigenetic mechanisms. In colorectal cancer, Ghorbani et al. identified differentially methylated genes involved in the Wnt and BMP signalling pathways, such as *WNT2*, *BMP2*, and *SFRP2*, which may serve as potential therapeutic targets [22]. In gastric cancer, Kim & Seo reported hypermethylation of *CDKN2A* and silencing of *MLH1* in Epstein-Barr virus-positive and MSI subtypes [56]. Lin et al. highlighted intratumoral epigenomic heterogeneity in HCC, while Liu et al. found that methylation of *SST* and *SH3GL2* may contribute to oesophageal cancer progression [43, 59]. Across cancers, aberrant methylation of tumour suppressors (e.g., *CDKN2A*, *MLH1*), histone modifications, and altered ncRNA expression (e.g., miRNAs) were consistently associated with tumour initiation, grade, and prognosis [13, 36, 54].

These findings are supported by broader evidence that aberrant DNA methylation of tumour suppressor genes (e.g., *CDKN2A*, *MLH1*), histone modifications, and altered non-coding RNA expression (e.g., miRNAs) have been implicated in tumour initiation and progression. For instance, hypermethylation of gene promoters in gastric cancer and HCC correlates with advanced tumour grade and poor prognosis [58]. Moreover, studies suggest that these alterations occur early in tumorigenesis, making them promising targets for therapeutic intervention, particularly if they affect key gene regulation pathways or are reversible through epigenetic therapies [60]**.**

The high degree of intra-tumoral heterogeneity observed in DSCs complicates both diagnosis and treatment. Multi-region sequencing studies in HCC and CRC reveal substantial inter-lesional and intra-lesional genomic divergence, including private subclonal mutations that may contribute to drug resistance and disease relapse [41, 43]. Understanding clonal evolution and heterogeneity at the single-cell level could facilitate more precise therapeutic strategies and prevent clonal escape under treatment pressure. Studies such as Xue et al*.* (2016) have documented the spatial and clonal heterogeneity of primary and metastatic lesions in HCC, highlighting the need for personalized therapeutic approaches [41].

Although germline alterations have been most frequently studied in CRC, relatively few studies have extended this focus to other DSC subtypes, limiting the current understanding of hereditary cancer risk across the broader DSCs spectrum. Significant gaps persist in the characterization of germline mutations linked to hereditary gastrointestinal cancer syndromes, such as Lynch Syndrome and familial intestinal gastric cancer, despite advances in tumour profiling. Table 1 highlights that rarer DSC subtypes such as gallbladder, ampullary, and small intestinal cancers were infrequently represented, reflecting both underfunding and limited sample availability. These underrepresented cancers may harbour distinct genomic profiles that remain poorly understood, highlighting the need for future studies to investigate their molecular characteristics. Expanding access to large-scale population biobanks, such as the UK Biobank, in conjunction with integrative genomic-phenomic analyses, may uncover novel insights into inherited cancer susceptibility, gene-environment interactions, and ancestry-specific risk variants [61]. The potential of biobanks to identify new cancer susceptibility loci has already been demonstrated, as seen in studies like Law et al*.* (2019), reinforcing their value in DSCs research [9].

An important consideration in interpreting these findings is the biological heterogeneity across DSCs. These malignancies arise from anatomically and histologically distinct tissues, including squamous and glandular epithelium, and therefore exhibit divergent molecular profiles [53, 54]. For example, oesophageal squamous cell carcinoma and oesophageal adenocarcinoma are known to differ substantially in their mutational landscape and underlying pathogenic mechanisms [53]. While this scoping review aimed to synthesize molecular evidence across DSCs, we have stratified findings by cancer subtype where possible to minimize overgeneralization. Nevertheless, pooling data across tumour types may obscure subtype-specific alterations, and future studies should prioritize more granular, histology-driven analyses [13, 58]. Where data permitted, findings were interpreted in a subtype-specific manner (e.g., oesophageal squamous cell carcinoma vs adenocarcinoma, and pancreatic ductal adenocarcinoma vs neuroendocrine tumours) to minimize biological oversimplification.

While the included studies provided valuable insights into the molecular features of DSCs, several limitations must be acknowledged. First, the evidence base comprised heterogeneous published studies with variable and often small cohort sizes, which limits statistical power, cross-study comparability, and generalizability of the findings. Furthermore, several studies lacked longitudinal clinical data or outcome measures, making it difficult to draw robust conclusions about the prognostic or therapeutic relevance of the identified molecular alterations. The synthesis was also constrained by methodological heterogeneity across studies, including differences in sequencing platforms, analytical pipelines, and reporting standards. The search also did not include grey literature databases, which may have excluded relevant studies. In some instances, authors specifically noted the absence of clinical actionability, meaning that while molecular aberrations were detected, their implications for treatment selection or patient management remained unclear. Importantly, most included studies did not incorporate independent validation cohorts or functional confirmation of reported molecular findings, which restricts confidence in the reproducibility and clinical applicability of the observations. These limitations underscore the importance of designing future studies that integrate molecular profiling with detailed clinical annotation and that include larger, more diverse patient populations.

Recent advances in single-cell sequencing and spatial transcriptomics have further refined our understanding of tumour heterogeneity in DSCs [75–77]. These approaches have revealed tumour subclones, lineage hierarchies, and microenvironmental interactions that are not captured by bulk sequencing, further emphasising the complexity of DSC molecular pathogenesis. Emerging studies have demonstrated that intra-tumour heterogeneity at the single-cell level contributes to therapeutic resistance, immune evasion, and disease progression [78, 79]. Incorporating such high-resolution approaches into future DSC research will be critical for delineating cell-type-specific molecular alterations and improving the precision of targeted therapeutic strategies.

Despite their potential clinical impact, germline mutations remain underexplored in DSC research. This is partly due to the predominant focus on sporadic cancers, limited availability of matched germline-tumour datasets, and the underdiagnosis of hereditary syndromes in clinical settings. Future research should prioritize efforts to integrate germline analysis alongside somatic profiling, particularly in younger patients and those with strong family histories, to enhance risk stratification and guide precision prevention strategies.

Integrating molecular data into clinical practice holds significant promise for improving the management of DSCs. Although targeted therapies based on actionable mutations like *KRAS G12C* and *ERBB2* are emerging [62], inconsistent molecular testing and inequitable access hinder clinical application. Future research should prioritize functional validation of emerging molecular alterations to advance translation, particularly in understudied DSC subtypes. Incorporating multi-omics approaches can further clarify the molecular drivers of tumour progression and treatment resistance. Prospective studies linking molecular profiles with treatment response, and expanding profiling in underrepresented populations, are critical to improving generalizability and reducing disparities. These efforts, supported by collaborative infrastructure and standardized molecular diagnostics, will be key to realizing the full potential of precision medicine in DSCs and improving outcomes through more personalized care.

## Conclusion

This scoping review provides a comprehensive synthesis of current literature on the molecular pathogenesis of DSCs from 2013 to 2025, encompassing 57 studies that applied NGS to uncover genetic, epigenetic, and transcriptional alterations across a spectrum of DSCs. While recurrent mutations such as *TP53*, *PIK3CA*, and *CDKN2A* were commonly reported across CRC, gastric, liver, and pancreatic cancers, significant knowledge gaps remain. The inclusion of recent studies published up to 2025 further underscores the evolving landscape of molecular characterisation in DSCs, particularly in relation to germline variants and emerging genomic technologies.

Research has been disproportionately focused on colorectal and gastric cancers, whereas oesophageal, gallbladder, ampullary, and other rare subtypes remain neglected despite harbouring distinctive molecular features. Although germline variants have been reported in colorectal, pancreatic, and fibrolamellar liver cancers, they remain underexplored in gastric and oesophageal cancers. Most included studies were retrospective, cross-sectional, and geographically clustered, which limits causal inference and generalisability. Moreover, despite the identification of actionable targets, inconsistent molecular testing practices and inequitable access to advanced diagnostics and targeted therapies continue to hinder translation of these discoveries into clinical precision oncology.

To address these limitations, larger multi-institutional, multi-omic studies are urgently needed, with systematic inclusion of rare digestive system cancers and underrepresented populations. Linking genomic data with clinical outcomes will be essential for accelerating translation and ultimately improving patient care in DSCs.

## Supplementary Information


Additional file 1.
Additional file 2.
Additional file 3.


## Data Availability

No primary datasets were generated or analysed during this study. This scoping review was conducted using published literature retrieved from bibliographic databases (PubMed/MEDLINE, Scopus, and Web of Science). All data supporting the findings of this study are available within the article, its supplementary materials, and the included references. The full search strategy and data extraction framework are provided in the supplementary information.
