# Supplementary material for: A systematic scoping review of the molecular pathogenesis of digestive system cancers (2013–2025)
Source: Discov Oncol. 2026 Jun 19;17:1266. doi: 10.1007/s12672-026-05374-6 (PMC13526742; doi:10.1007/s12672-026-05374-6)
Supplement: Supplementary file 1 [file 12672_2026_5374_MOESM1_ESM.pdf]

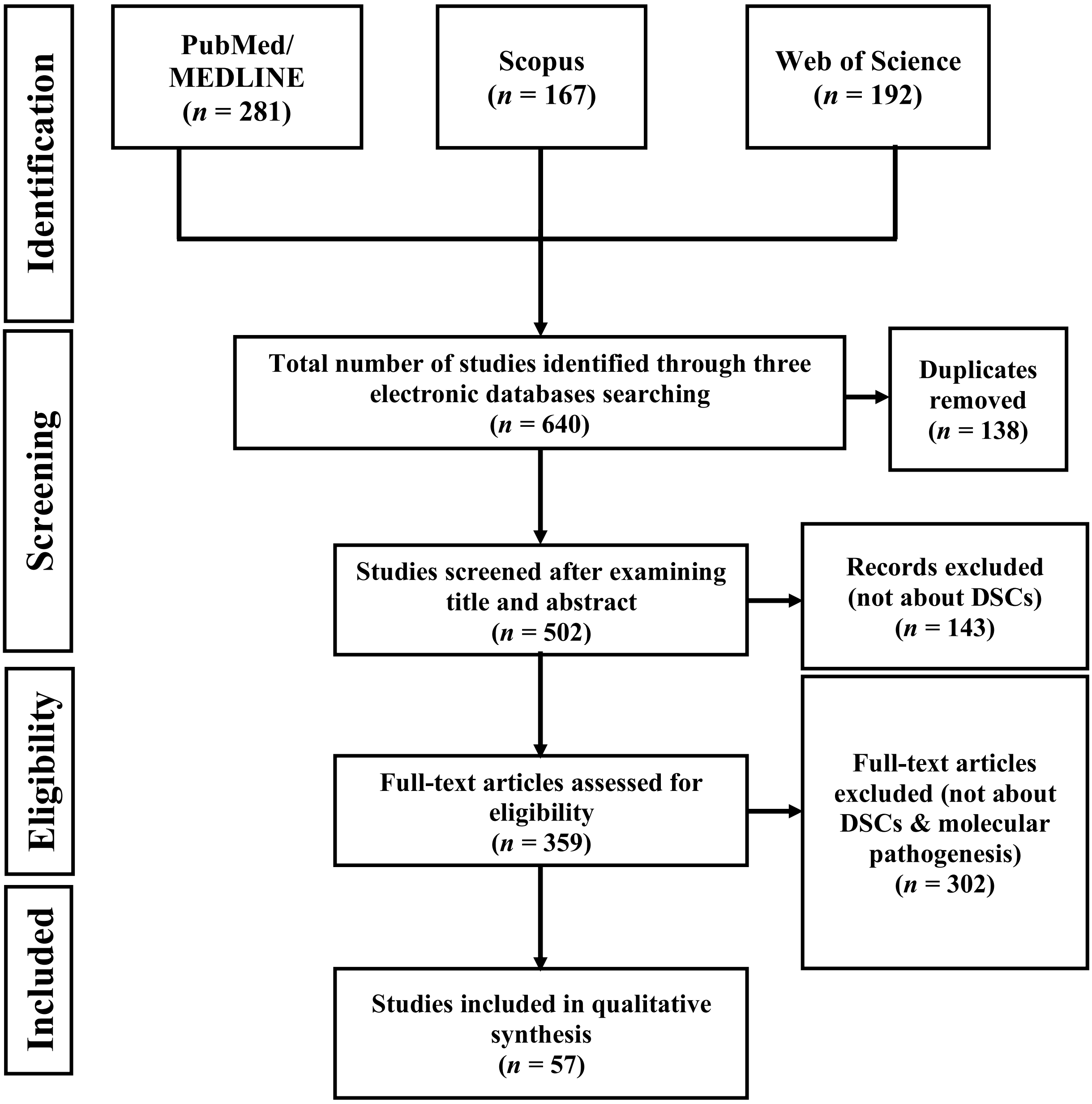

Figure 1. Preferred Reporting Items for Systematic Reviews and Meta-Analyses (PRISMA) flow diagram for scoping review on the landscape of molecular pathogenesis
