# Supplementary material for: A systematic scoping review of the molecular pathogenesis of digestive system cancers (2013–2025)
Source: Discov Oncol. 2026 Jun 19;17:1266. doi: 10.1007/s12672-026-05374-6 (PMC13526742; doi:10.1007/s12672-026-05374-6)
Supplement: Supplementary file 2 [file 12672_2026_5374_MOESM2_ESM.pdf]

Molecular alteration pathway	Colorectal	Gastric	Liver	Pancreatic	Oesophageal	Gallbladder
**TP53**	●●●	●●●	●●●	●●●	●●●	●●●
**KRAS**	●●●	●	●	●●●	●	●●
**APC / Wnt signalling**	●●●	●	●●●	●	–	●
**PI3K/AKT (PIK3CA/PTEN)**	●●●	●●●	●	●	●	●
**MMR / MSI pathway**	●●●	●●	●	●	●	–
**ERBB signalling (ERBB2/EGFR)**	●	●●●	●	●	●●	●●●
**DNA damage repair**	●●	●	●	●●●	●	●
**Chromatin remodelling (e.g. ARID1A)**	●	●●●	●●	●	●	●
**RTK–RAS–MAPK signalling**	●●●	●●●	●●●	●●●	●●●	●●●
**Epigenetic dysregulation**	●●	●●●	●	●	●●	●
**Inflammation / viral-associated pathways**	●	●	●●●	●	●	●
**Mucin/ECM/glycosylation pathways**	●	●	●●	●	–	●

Figure 2. Comparative overview of recurrent molecular alterations and pathways across digestive system cancers.

This figure summarises commonly reported molecular alterations and biological pathways identified across the included studies. The qualitative scale reflects the relative consistency of reporting across studies rather than quantitative mutation frequency. ●●● indicates alterations consistently reported across multiple studies, ●● indicates moderate reporting, ● indicates less frequent or context-specific reporting, and – indicates limited or no prominent reporting within the included literature. Abbreviations: MMR, mismatch repair; MSI, microsatellite instability; ECM, extracellular matrix.
