# Supplementary material for: A systematic scoping review of the molecular pathogenesis of digestive system cancers (2013–2025)
Source: Discov Oncol. 2026 Jun 19;17:1266. doi: 10.1007/s12672-026-05374-6 (PMC13526742; doi:10.1007/s12672-026-05374-6)
Supplement: Supplementary file 3 [file 12672_2026_5374_MOESM3_ESM.pdf]

Supplementary Methods: Search strategy
Searches were conducted on July 7, 2025

1. **PubMed/MEDLINE (271)**

Search	Description
#1	((((((((((((((((((gastrointestinal cancer[MeSH Terms]) OR (gastrointestinal cancers[MeSH Terms])) OR (cancer of gastrointestinal tract[MeSH Terms])) OR (cancer of the gastrointestinal tract[MeSH Terms])) OR (digestive system neoplasm[MeSH Terms])) OR (digestive system neoplasms[MeSH Terms])) OR (cancer of digestive system[MeSH Terms])) OR (gastric cancer[MeSH Terms])) OR (gastric cancers[MeSH Terms])) OR (esophageal cancer[MeSH Terms])) OR (esophageal cancers[MeSH Terms])) OR (liver cancer[MeSH Terms])) OR (liver cancers[MeSH Terms])) OR (colorectal cancer[MeSH Terms])) OR (colorectal cancers[MeSH Terms])) OR (colon cancer[MeSH Terms])) OR (colon cancers[MeSH Terms])) OR (pancreatic cancer[MeSH Terms])) OR (pancreatic cancers[MeSH Terms])
#2	((((molecular changes[Title/Abstract]) OR (genetic changes[Title/Abstract])) OR (pathogenesis[Title/Abstract])) OR (molecular pathology[MeSH Terms])) OR (molecular pathologies[MeSH Terms])
#3	(((High-Throughput Nucleotide Sequencing[MeSH Terms]) OR (sequence analysis, dna[MeSH Terms])) OR (next generation sequencing[MeSH Terms])) OR (NGS[Title/Abstract])
**Final Search**	#1 AND #2 AND #3

Limiter:
Published Date: 10 years

2. Scopus (154)

Search documents	Search within
"molecular changes" OR "genetic changes" OR "pathogenesis" OR "molecular pathology" OR "molecular pathologies"	Article title, Abstract, Keywords
AND	
"High-Throughput Nucleotide Sequencing" OR "sequence analysis, dna" OR "next generation sequencing" OR "NGS"	Article title, Abstract, Keywords
AND	
"gastrointestinal cancer" OR "gastrointestinal cancers" OR "cancer of gastrointestinal tract" OR "cancer of the gastrointestinal tract" OR "digestive system neoplasm" OR "digestive system neoplasms" OR "cancer of digestive system" OR "gastric cancer" OR "gastric cancers" OR "esophageal cancer" OR "esophageal cancers" OR "liver cancer" OR "liver cancers" OR "colorectal cancer" OR "colorectal cancers" OR "colon cancer" OR "colon cancers" OR "pancreatic cancer" OR "pancreatic cancers"	Article title, Abstract, Keywords

Limiters:
Published Date: 10 years
Document type: Original and review articles
Language: English
Keyword: High Throughput Sequencing

3. **Web of Science (WoS) Core Collection (178)**
https://www.webofscience.com/wos/woscc/summary/3c1c0562-3130-474a-8402-6db0a7e66301-80cdf57f/relevance/1

**Search**	**Query**
**#1**	(TS=(("gastrointestinal cancer" OR "gastrointestinal cancers" OR "cancer of gastrointestinal tract" OR "cancer of the gastrointestinal tract" OR "digestive system neoplasm" OR "digestive system neoplasms" OR "cancer of digestive system" OR "gastric cancer" OR "gastric cancers" OR "esophageal cancer" OR "esophageal cancers" OR "liver cancer" OR "liver cancers" OR "colorectal cancer" OR "colorectal cancers" OR "colon cancer" OR "colon cancers" OR "pancreatic cancer" OR "pancreatic cancers"))
**#2**	TS=("molecular changes" OR "genetic changes" OR "pathogenesis" OR "molecular pathology")
**#3**	TS=("High-Throughput Nucleotide Sequencing" OR "sequence analysis, dna" OR "next generation sequencing" OR "NGS"))
**Final Search**	#1 AND #2 AND #3

Limiters:
Published Date: 10 years (2013-2023)
Document type: Article and review articles
Language: English

WOS search term:
(TS=(("gastrointestinal cancer" OR "gastrointestinal cancers" OR "cancer of gastrointestinal tract" OR "cancer of the gastrointestinal tract" OR "digestive system neoplasm" OR "digestive system neoplasms" OR "cancer of digestive system" OR "gastric cancer" OR "gastric cancers" OR "esophageal cancer" OR "esophageal cancers" OR "liver cancer" OR "liver cancers" OR "colorectal cancer" OR "colorectal cancers" OR "colon cancer" OR "colon cancers" OR "pancreatic cancer" OR "pancreatic cancers")) AND TS=("molecular changes" OR "genetic changes" OR "pathogenesis" OR "molecular pathology") AND TS=("High-Throughput Nucleotide Sequencing" OR "sequence analysis, dna" OR "next generation sequencing" OR "NGS"))
